# Effects of the abdominal fat distribution on the relationship between exposure to air pollutants and thyroid hormones among Korean adult males

**DOI:** 10.1186/s40001-023-01394-y

**Published:** 2023-10-11

**Authors:** Hyun-Jin Kim, Byungmi Kim, Seyoung Kim, Hyuktae Kwon, Jae Moon Yun, Belong Cho, Jin-Ho Park

**Affiliations:** 1 National Cancer Control Institute, National Cancer Center, Goyang, 10408 South Korea; 2 Department of Family Medicine, Seoul National University Hospital, 03 Daehakro, Yeongun-Dong, Jongno-Gu, Seoul, 03080 Korea; 3 Department of Family Medicine, Seoul National University College of Medicine, 103 Daehakro, Yeongun-Dong, Jongno-Gu, Seoul, 03080 Korea

**Keywords:** Air pollution, Thyroid function, Abdominal fat distribution, General population

## Abstract

**Background:**

Several significant associations between air pollution and thyroid function have been reported, but few studies have identified whether these associations differ by obesity, particularly its regional distribution. We assessed the relationship between ambient air pollution and thyroid hormone, and whether this relationship is modified by abdominal adiposity, as indicated by the waist circumference, visceral adipose tissue (VAT), subcutaneous adipose tissue (SAT), and visceral-to-subcutaneous fat ratio (VSR) in Korean men.

**Methods:**

We included 2440 male adults in the final analysis and used each person’s annual average exposure to four air pollutants: particulate matter with an aerodynamic diameter ≤ 10 μm (PM_10_), nitrogen dioxide, sulfur dioxide (SO_2_), and carbon monoxide (CO). Abdominal fat deposition was quantified by computed tomography. Serum thyrotropin (TSH) and free thyroxine (FT_4_) concentrations were measured for thyroid hormone. To evaluate the relationship between air pollution and thyroid hormone according to adiposity, we performed multiple linear regression analysis on the two subgroups stratified by abdominal fat level.

**Results:**

Abdominal adiposity was significantly related to FT_4_ concentration. The exposures to air pollutants were associated with increased TSH and decreased FT_4_ concentrations. In stratified analysis using abdominal fat traits, ambient air pollution except for SO_2_ was significantly related to increased TSH and decreased FT_4_ concentrations in the high adiposity group (all *p* < 0.05), but not in the normal adiposity group. Among the air pollutants, PM_10_ showed an association with an increase of TSH concentration in all group with high adiposity, including high VAT, high SAT, and high VSR groups (all *p* < 0.05). In case of FT_4_, CO showed a similar pattern. Among the abdominal fat-related traits, the VSR in the high adiposity group had the largest effect on the relationship between exposure to air pollutants and thyroid hormone.

**Conclusions:**

This study suggests the first clue that the relationship between air pollution exposure and thyroid hormone differs according to abdominal fat distribution among Korean adult males.

## Background

Ambient air pollution contains known human carcinogens and is a problem worldwide, and has recently become a major public concern in Korea [1]. A report from the Organization for Economic Cooperation and Development (OECD) documented that outdoor air pollution in South Korea was estimated at 359 premature deaths per million individuals in 2010. If this situation continues, it will rise to more than 1100 per million individuals by 2060 [2]. Most premature deaths related to air pollution were linked to cardiovascular diseases, including stroke and myocardial infarction [3, 4].

In recent years, emerging evidence has shown the harmful effects of ambient air pollution on thyroid hormone levels [5–9]. Experimental findings have shown how their toxic effects on environmental chemicals affect thyroid hormone levels [10, 11]. A more recent study has suggested that obese status mediates the association between ambient air pollution and thyroid function [1]. Although the mechanisms underlying this relationship are not understood completely, several possible hypotheses have been proposed. Obese status, especially abdominal obesity, is linked to the thyroid functions related to oxidation of fatty acids, lipid metabolism, and so on [12, 13]. Abdominal fat distribution, as measured by the subcutaneous and visceral fat area, plays a role in the high risk of diseases, including metabolic diseases, cardiovascular diseases, and hypertension [14, 15].

It is essential to understand the role of regional abdominal fat traits to understand these associations and mechanisms more fully. Excessive visceral adipose tissue (VAT) may contribute to elevated oxidative stress or oxidative damage [16, 17]. VAT secretes large amounts of cytokines that induce inflammation, including tumor necrosis factor-α (TNF-α) and interleukin-6 (IL-6), compared to subcutaneous fat [18, 19]. In fact, a previous study suggested an increase in VAT as the best predictor of thyrotropin (TSH) in obesity [20]. By contrast, subcutaneous fat may affect the change of thyroid hormone in specific tissue [21]. The thyroid hormone receptor is also more activated in subcutaneous adipose tissue (SAT) than in VAT [22].

In this context, we reasoned that abdominal fat may mediate the relationship between air pollution and thyroid hormone and that this may be reflected in differences according to adiposity traits. However, previous studies of the effects of obesity on the relationship between environmental pollutants and thyroid hormones have mostly used body mass index (BMI) as a proxy variable for obesity [1, 23]. This indirect indicator of obesity cannot accurately distinguish certain depots of abdominal fat. Therefore, the precise estimation of fat mass using computed tomography (CT) is essential to examine these potential associations further. To our knowledge, no studies have evaluated this hypothesis. In this study, we assessed the relationship of exposure to air pollutants and thyroid hormone in Korean adults and whether these relationships are mediated by abdominal fat type.

## Methods

### Participants

The subjects included in our study were recruited at two health screening hospitals run by a university Hospital in Seoul from 2009 to 2015, (1) the Seoul National University Hospital Health Promotion Center (site A), and (2) the Seoul National University Hospital Healthcare System Gangnam Center (site B). Korean adults receive regular health examinations for the prevention or early diagnosis of illness. There is a large difference in the abdominal fat mass between both sexes, and the accumulation of visceral fat tissue in Korean adults is more pronounced in men than in women [24, 25]. For this reason, mainly adult men were selected and the proportion of women was very low (*n* = 2560 and 344 for men and women, respectively). In the analyses, when we classified the subjects as two groups by adiposity level (normal vs. high), the difference in the abdominal adiposity distribution between male and female made it difficult to apply identical cutoff points. Therefore, we included only adult men in this study.

A total of 2560 adults underwent the physical examinations, including abdominal CT, during the period noted above. We excluded 120 who met the following exclusion criteria from the final analysis: (1) those whose air pollution exposure concentration could not be estimated because of missing postal code information; (2) those with missing information for thyroid-related outcomes, such as serum TSH and free thyroxine (FT_4_) concentrations; (3) those whose these hormone levels could not be measured accurately because of thyroid surgery or medications that may affect thyroid hormones; and (4) those whose lifestyle data such as frequency of alcohol use, smoking status, and moderate activity were incomplete. A total of 2440 adults were used for the final statistical analysis.

The need for informed consent was waived by our institutional review board, because this was a retrospective study using de-identified data. The protocol for this study was approved by the institutional review boards of the Seoul National University Hospital and National Cancer Center.

### Assessment of the exposure to air pollution

Atmospheric monitoring data to obtain 24-h concentrations were obtained from a total of 328 nationwide monitoring stations operated by the Ministry of the Environment of South Korea. The data included real-time measurements of various air pollutants, including particulate matter with an aerodynamic diameter of ≤ 10 μm (PM_10_), sulfur dioxide (SO_2_), nitrogen dioxide (NO_2_), and carbon monoxide (CO). Each participant’s residential postal code was extracted from the internal hospital database to estimate exposure levels corresponding to each individual. We checked the monitoring point nearest to each participant’s residence using the postal code information and calculated the annual average values of all air pollutants. The individual exposure concentration was estimated by matching the annual mean concentration based on the medical examination year at the monitoring site closest to the residence of each individual. Of the total monitoring sites, 302 monitoring subsets were finally used. The concentrations of all four air pollutants were interrelated, and the correlation levels ranged from 0.16 to 0.69 (data not shown).

### Assessment of the abdominal adiposity

Obesity indicators, including several abdominal fat-related traits, were measured when individuals visited for health checkups. Waist circumference (WC) was measured with the subject wearing thin clothing. We classified the subjects into two groups according to the criteria for abdominal obesity in Asian men as follows: normal (WC < 90 cm) and obese (WC ≥ 90 cm). We measured abdominal adiposity using a CT scanner (Somatom Sensation 16 CT scanner, Siemens AG, Erlangen, Germany). The cross-sectional area of each adipose tissue site, such as VAT and SAT, was calculated using Rapidia software (version 2.8; Infinitt, Seoul, South Korea). The visceral fat area was estimated by drawing a boundary with the parietal peritoneum or transversalis fascia, except for the vertebrae and spinal muscles. The subcutaneous fat area was defined by deducting the visceral fat area from the total fat area, excluding the vertebrae and spinal muscles. We included the visceral-to-subcutaneous fat ratio (VSR). Participants were divided into two groups (normal adiposity and high adiposity) using a cutoff criterion of 100 cm^2^ for VAT and SAT. For the VSR, a cutoff criterion of 1.0 was used.

### Assessment of thyroid function and other variables

We obtained data to evaluate the thyroid hormone level from standard blood tests. Thyroid hormone included serum TSH and FT_4_ concentrations. TSH and FT_4_ were measured using immunoradiometric assay kit and radioimmunoassay kits (RIAKEY, Shinjin Medics Inc. Seoul, Korea), respectively. The manufacturer reference ranges for TSH and FT_4_ were 0.3–5.0 μIU/mL and 0.7–1.8 ng/dL, respectively. To test the associations between exposure to air pollution and thyroid hormones, we controlled the analysis for potential confounding variables, including the site of recruitment, age, status of alcohol use (never, former, or current drinker), cigarette smoking (never, former, or current smoker), and physical activity (yes or no). Physical activity was assessed with the short form of the International Physical Activity Questionnaires (IPAQ), and was defined as engaging moderate (i.e., carrying light loads, bicycling at a regular pace, or doubles tennis) or vigorous intensity activities (i.e., heavy lifting, digging, aerobics, or fast bicycling) for at least 10 min a week. Lifestyle behaviors were assessed using questionnaires on the day of the medical examination.

### Statistical analysis

The distribution of TSH and FT_4_ concentrations were checked prior to analyses of association. Because both TSH and FT_4_ followed a nonnormal distribution, we used the best transformation approaches, and the square root and natural logarithm transformations were applied to the TSH and FT_4_ levels, respectively. To test the difference in participant characteristics between groups according to each abdominal fat level, a *t* test and a Chi-square test were performed according to the type of each variable (i.e., continuous or categorical variables). We considered multiple linear regression analysis to assess the relationship between air pollution exposure and thyroid hormone. In our data, the correlations between all four air pollutants were significant, even though the value of variance inflation factor for multicollinearity was less than 10. Therefore, we finally did not include other air pollutants as confounders in our model. In adjusted model, the results of the association analyses were controlled for the recruitment center, age, smoking status, status of alcohol use, and physical activity [*Y* = *β*_0_ + *β*_1_ recruitment center + *β*_2_ age + *β*_3_ smoking status + *β*_4_ status of alcohol use + *β*_5_ physical activity + *β*_6_ each air pollutant + *e*]. We also conducted a stratified analysis for the two subgroups according to abdominal fat traits, including WC, VAT, SAT, and VSR. Beta coefficients (*β*) and standard errors (SEs) were converted to the interquartile range (IQR) (9.1 μg/m^3^ for PM_10_, 13.8 ppb for NO_2_, 1.5 ppb for SO_2_, and 0.2 ppm for CO). All statistical analyses were performed using SAS (version 9.4; SAS Institute, Cary, NC, USA).

## Results

### Characteristics of participants

The characteristics of the 2440 participants included in the final analyses are presented in Table 1, and the results are shown for each group according to each abdominal fat level. For each type of abdominal fat, the number of subjects with high SAT (*n* = 1897) was higher than that of high VAT (*n* = 1762) or high VSR (*n* = 1061). In the results for VAT, the mean values of age, BMI, and WC were greater in the group with high VAT than in the normal group. The proportion of former- or current-smokers (80.1%) and -drinkers (87.5%) in the high VAT group was higher than in the normal VAT group (71.5% and 82.7% for former- or current-smokers and -drinkers, respectively). In addition, more participants responded “yes” to physical activity in the normal groups, compared to high VAT group. In the case of the SAT or VSR, the results were similar to those of the VAT. However, the differences in smoking and alcohol drinking status between the two groups for SAT were not statistically significant (both *p* > 0.05). For VSR, there was no statistical difference in physical activity between the two groups (*p* = 0.1291). In relation to thyroid hormone, statistically significant difference was only identified in FT_4_ level between the two groups for VAT (*p* = 0.0071). The mean values of four air pollutants were 48.9 μg/m^3^ for PM_10_, 31.0 ppb for NO_2_, 4.9 ppb for SO_2_, and 0.6 ppm for CO, respectively.

**Table 1:** Characteristics of study participants according to each abdominal adiposity group

Characteristics	Groups according to abdominal adiposity level								
	VAT			SAT			VSR		
	Normal (VAT < 100cm^2^)	High (VAT ≥ 100cm^2^)	*p*	Normal (SAT < 100cm^2^)	High (SAT ≥ 100cm^2^)	*p*	Normal (VSR < 1)	High (VSR ≥ 1)	*p*
n	678	1762		543	1897		1379	1061	
Site of recruitment			0.0135			0.2795			0.0287
Site A	553 (81.6)	1356 (77.0)		434 (79.9)	1475 (77.8)		1101 (79.8)	808 (76.2)	
Site B	125 (18.4)	406 (23.0)		109 (20.1)	422 (22.2)		278 (20.2)	253 (23.9)	
Age (years)	48.8 (8.2)	50. 8 (7.8)	< 0.0001	51.1 (8.1)	50.0 (7.9)	0.0038	48.5 (8.0)	52.5 (7.3)	< 0.0001
Smoking			< 0.0001			0.2198			0.0075
Never	193 (28.5)	352 (20.0)		136 (25.1)	409 (21.6)		334 (24.2)	211 (19.9)	
Former-smokers	246 (36.3)	785 (44.6)		219 (40.3)	812 (42.8)		549 (39.8)	482 (45.4)	
Current-smokers	239 (35.2)	625 (35.5)		188 (34.6)	676 (35.6)		496 (36.0)	368 (34.7)	
Alcohol drinking			0.0002			0.3645			0.0002
Never	117 (17.3)	221 (12.5)		81 (14.9)	257 (13.6)		220 (16.0)	118 (11.1)	
Former-drinkers	59 (8.7)	105 (6.0)		42 (7.7)	122 (6.4)		105 (7.6)	59 (5.6)	
Current-drinkers	502 (74.0)	1436 (81.5)		420 (77.4)	1518 (80.0)		1054 (76.4)	884 (83.3)	
Height (cm)	170.2 (6.1)	170.7 (5.9)	0.0864	169.1 (5.9)	171.0 (5.9)	< 0.0001	171.2 (6.1)	169.6 (5.6)	< 0.0001
Weight (kg)	64.7 (7.8)	74.3 (9.3)	< 0.0001	62.6 (6.8)	74.2 (9.1)	< 0.0001	71.8 (10.4)	71.5 (9.2)	0.4706
BMI (kg/m^2^)	22.3 (2.2)	25.5 (2.5)	< 0.0001	21.9 (2.0)	25.4 (2.5)	< 0.0001	24.4 (3.0)	24.8 (2.6)	0.0009
WC (cm)	81.0 (6.1)	91.1 (6.6)	< 0.0001	80.0 (5.8)	90.7 (6.7)	< 0.0001	87.7 (8.3)	89.1 (7.2)	< 0.0001
Physical activity			0.0052			0.0353			0.1291
Yes	391 (57.7)	905 (51.4)		310 (57.1)	986 (52.0)		751 (54.5)	545 (51.4)	
No	287 (42.3)	857 (48.6)		233 (42.9)	911 (48.0)		628 (45.5)	516 (48.6)	
Thyroid hormones									
TSH (μIU/mL)	1.8 (1.1)	1.8 (1.0)	0.9422	1.8 (1.0)	1.8 (1.0)	0.6276	1.8 (1.0)	1.8 (1.1)	0.7824
FT_4_ (ng/dL)	1.4 (0.3)	1.3 (0.2)	0.0071	1.4 (0.3)	1.4 (0.3)	0.4147	1.4 (0.3)	1.3 (0.3)	0.1197

VAT, visceral adipose tissue; SAT, subcutaneous adipose tissue; VSR, visceral-to-subcutaneous fat ratio; BMI, body mass index; WC, waist circumference; TSH, Thyroid Stimulating Hormone; FT_4_, Free Thyroxine

Data are provided as means (standard deviations) for continuous variables, such as age, height, weight, BMI, WC, and thyroid hormones

TSH and FT_4_ levels were transformed to square root and natural logarithms for the *t* test on thyroid hormones, respectively

### Relationship between adiposity traits or air pollutants and thyroid hormones

We investigated the relationship between adiposity-related traits or exposure to four air pollutants and thyroid hormones, such as TSH and FT_4_ levels (Table 2). None of the adiposity traits were significantly related to TSH concentration (all *p* > 0.05). FT_4_ concentration was significantly associated with the adiposity traits WC, VAT, and SAT (all *p* < 0.05) but not to the VSR. Increases in IQR for PM_10_, NO_2_, and CO exposures were closely related to the increase in TSH concentration when analyzed using the square root scale (all *p* < 0.05). Only NO_2_ was significantly related to a low FT_4_ concentration (*p* in the adjusted model = 0.0147).

**Table 2:** Regression results for the association between exposure to abdominal adiposity, air pollution, and thyroid related traits

	TSH (μIU/mL)				FT_4_ (ng/dL)			
	Crude Model		Adjusted Model^a^		Crude Model		Adjusted Model^a^	
	*β (SE)*	*p*	*β (SE)*	*p*	*β (SE)*	*p*	*β (SE)*	*p*
Adiposity trait								
WC (cm)	0.0013 (0.0009)	0.1762	0.0014 (0.0009)	0.1368	− 0.0021 (0.0005)	< 0.0001	− 0.0022 (0.0005)	< 0.0001
VAT (cm^2^)	0.0055 (0.0128)	0.6691	0.0159 (0.0131)	0.2241	− 0.0226 (0.0062)	0.0003	− 0.0208 (0.0063)	0.0010
SAT (cm^2^)	0.0177 (0.0135)	0.1884	0.0107 (0.0136)	0.4325	− 0.0141 (0.0065)	0.0301	− 0.0186 (0.0066)	0.0047
VSR	− 0.0093 (0.0089)	0.2931	− 0.0027 (0.0090)	0.7651	− 0.0038 (0.0043)	0.3737	− 0.0021 (0.0043)	0.6378
Air pollution								
PM_10_ (μg/m^3^)	0.0216 (0.0093)	0.0205	0.0256 (0.0096)	0.0077	− 0.0058 (0.0045)	0.2009	− 0.0056 (0.0047)	0.2288
NO_2_ (ppb)	0.0203 (0.0080)	0.0114	0.0190 (0.0081)	0.0196	− 0.0084 (0.0039)	0.0293	− 0.0097 (0.0040)	0.0147
SO_2_ (ppb)	− 0.0003 (0.0084)	0.9701	− 0.0013 (0.0084)	0.8762	− 0.0001 (0.0041)	0.9753	− 0.0003 (0.0041)	0.9443
CO (ppm)	0.0259 (0.0106)	0.0150	0.0266 (0.0107)	0.0129	− 0.0080 (0.0051)	0.1218	− 0.0090 (0.0052)	0.0830

TSH, Thyroid Stimulating Hormone; FT_4_, Free Thyroxine; WC, waist circumference; VAT, visceral adipose tissue; SAT, subcutaneous adipose tissue; VSR, visceral-to-subcutaneous fat ratio; PM_10_, particulate matter ≤ 10 μm in diameter; NO_2_, nitrogen dioxide; SO_2_, sulfur dioxide; CO, carbon monoxide; SE, standard error

TSH and FT_4_ levels were transformed to square root and natural logarithms, respectively

The beta coefficient and standard error in adiposity measures including VAT and SAT was converted by scale to the 100 cm^2^ area

The beta coefficient and 95% confidence interval in each air pollutant was scaled to the interquartile range for each pollutant, respectively (9.1 μg/m^3^ for PM_10_, 13.8 ppb for NO_2_, 1.5 ppb for SO_2_, and 0.2 ppm for CO)

^a^The result was adjusted for site of recruitment, age, smoking status (never- or ex- vs. current-smokers), alcohol consumption (never- or ex- vs. current-drinkers), and physical activity (yes vs. no)

### Relationship between air pollutants and TSH according to adiposity groups

The association results between four air pollutants and thyroid hormone in two adiposity groups are shown in Table 3. In the stratified analysis of WC, relations between exposure to three air pollutants and TSH level differed between the two groups (high vs. normal WC). Compared with subjects with the normal adiposity, the high adiposity group showed positive associations between TSH concentration and exposure to all air pollutants excluding SO_2_ (all *p* < 0.05). In the case of VAT, PM_10_ was significantly related to an increase in the TSH concentration in the high VAT group (*p* for adjusted model = 0.322) but not in the normal VAT group (*p* for adjusted model = 0.1168). In the case of NO_2_ and SO_2_ exposures, there was no difference between the two groups, but CO exposure was significantly related to the TSH concentration in the normal VAT group. Similar patterns were observed for the SAT area. For VSR, exposure to PM_10_ (*p* in the adjusted model = 0.0407) and NO_2_ (*p* in the adjusted model = 0.0390) was significantly related to an increase in TSH concentration in the subjects with high VSR but not in those with normal VSR (both *p* > 0.05). By contrast, exposure to SO_2_ and CO was not significantly related to TSH concentration in either VSR group (all *p* > 0.05). Among the air pollutants, PM_10_ showed a distinct association with an increased TSH concentration in high VAT, high SAT, and high VSR groups, (all *p* < 0.05), but not in the subjects with normal adiposity (all *p* > 0.05).

**Table 3:** Results of stratified analyses by adiposity traits for the association between exposure to air pollution and thyroid stimulating hormone

Abdominal adiposity	Exposure	TSH (μIU/mL)							
		Normal adiposity				High adiposity			
		Crude Model		Adjusted Model^a^		Crude Model		Adjusted Model^a^	
		*β (SE)*	*p*	*β (SE)*	*p*	*β (SE)*	*p*	*β (SE)*	*p*
WC (cm)	Sample *n*	WC < 90 cm (*n* = 1016)				WC ≥ 90 cm (*n* = 1424)			
	PM_10_ (μg/m^3^)	0.0088 (0.0139)	0.5279	0.0202 (0.0145)	0.1636	0.0323 (0.0125)	0.0098	0.0301 (0.0128)	0.0193
	NO_2_ (ppb)	0.0116 (0.0123)	0.3474	0.0139 (0.0125)	0.2665	0.0275 (0.0105)	0.0093	0.0229 (0.0107)	0.0323
	SO_2_ (ppb)	0.0045 (0.0128)	0.7247	0.0028 (0.0128)	0.8271	− 0.0027 (0.0112)	0.8118	− 0.0004 (0.0111)	0.9736
	CO (ppm)	0.0057 (0.0165)	0.7317	0.0076 (0.0166)	0.6482	0.0403 (0.0139)	0.0038	0.0391 (0.0139)	0.0051
VAT (cm^2^)	Sample *n*	VAT < 100 cm^2^ (*n* = 678)				VAT ≥ 100 cm^2^ (*n* = 1762)			
	PM_10_ (μg/m^3^)	0.0369 (0.0180)	0.0405	0.0286 (0.0182)	0.1168	0.0158 (0.0109)	0.1466	0.0242 (0.0113)	0.0322
	NO_2_ (ppb)	0.0348 (0.0148)	0.0190	0.02467 (0.0148)	0.0961	0.0141 (0.0095)	0.1398	0.0165 (0.0097)	0.0903
	SO_2_ (ppb)	− 0.0050 (0.0160)	0.7575	− 0.0019 (0.0157)	0.9018	0.0015 (0.0099)	0.8820	− 0.0001 (0.0099)	0.9905
	CO (ppm)	0.0537 (0.0203)	0.0084	0.0520 (0.0201)	0.0099	0.0151 (0.0125)	0.2262	0.0172 (0.0126)	0.1731
SAT (cm^2^)	Sample *n*	SAT < 100 cm^2^ (*n* = 543)				SAT ≥ 100 cm^2^ (*n* = 1897)			
	PM_10_ (μg/m^3^)	0.0374 (0.0207)	0.0719	0.0264 (0.0210)	0.2087	0.0174 (0.0104)	0.0953	0.0244 (0.0108)	0.0239
	NO_2_ (ppb)	0.0390 (0.0171)	0.0226	0.0296 (0.0169)	0.0804	0.0148 (0.0091)	0.1024	0.0161 (0.0093)	0.0834
	SO_2_ (ppb)	− 0.0016 (0.0167)	0.9239	0.0048 (0.0163)	0.7702	0.0004 (0.0098)	0.9676	− 0.0014 (0.0098)	0.8834
	CO (ppm)	0.0530 (0.0223)	0.0180	0.0509 (0.0219)	0.0203	0.0177 (0.0121)	0.1437	0.0196 (0.0122)	0.1085
VSR	Sample *n*	VSR < 1.0 (*n* = 1379)				VSR ≥ 1.0 (*n* = 1061)			
	PM_10_ (μg/m^3^)	0.0211 (0.0123)	0.0857	0.0230 (0.0127)	0.0698	0.0221 (0.0142)	0.1205	0.0302 (0.0147)	0.0407
	NO_2_ (ppb)	0.0156 (0.0108)	0.1464	0.0132 (0.0110)	0.2276	0.0259 (0.0120)	0.0313	0.0253 (0.0122)	0.0390
	SO_2_ (ppb)	− 0.0093 (0.0115)	0.4196	− 0.0100 (0.0116)	0.3859	0.0097 (0.0123)	0.4347	0.0067 (0.0122)	0.5870
	CO (ppm)	0.0211 (0.0142)	0.1390	0.0210 (0.0143)	0.1416	0.0320 (0.0161)	0.0462	0.0315 (0.0161)	0.0509

TSH, Thyroid Stimulating Hormone; WC, waist circumference; VAT, visceral adipose tissue; SAT, subcutaneous adipose tissue; VSR, visceral-to-subcutaneous fat ratio; PM_10_, particulate matter ≤ 10 μm in diameter; NO_2_, nitrogen dioxide; SO_2_, sulfur dioxide; CO, carbon monoxide; SE, standard error

TSH level was transformed to square root

The beta coefficient and 95% confidence interval in each air pollutant was scaled to the interquartile range for each pollutant, respectively (9.1 μg/m^3^ for PM_10_, 13.8 ppb for NO_2_, 1.5 ppb for SO_2_, and 0.2 ppm for CO)

^a^The result was adjusted for site of recruitment, age, smoking status (never- or ex- vs. current-smokers), alcohol consumption (never- or ex- vs. current-drinkers), and physical activity (yes vs. no)

### Relationship between air pollutants and FT_4_ according to adiposity groups

The associations between four air pollutants and FT_4_ level in groups with normal or high adiposity are shown in Table 4. In the stratified association of WC, compared to the normal group, CO exposure was significantly related to FT_4_ concentration in the high WC group (*p* for the adjusted model = 0.0443). However, other air pollutants, such as PM_10_, NO_2_, and SO_2_, was not associated with FT_4_ concentration in both adiposity groups (all *p* > 0.05). For VAT, PM_10_ exposure was not significantly related to FT_4_ concentration in the normal adiposity group (*p* for adjusted model = 0.2471). However, in high visceral adiposity groups, VAT area was significantly associated with a low FT_4_ concentration (*p* for the adjusted model = 0.0275). Similarly, the associations between FT_4_ levels and NO_2_ (*p* for the adjusted model = 0.0004) and CO exposure (*p* for adjusted model = 0.0249) were significant only in the subjects with the high VAT area and not in the normal VAT group. This pattern was not observed for SO_2_ exposure. In the analysis of SAT, NO_2_ (*p* for the adjusted model = 0.0064) exposure and CO (*p* for the adjusted model = 0.0467) exposure were related to lower FT_4_ concentration, especially in the high SAT group. IQR increases in NO_2_ and CO exposure were related to decreases of 0.0121 mIU/L (SE = 0.0044) and 0.0116 mIU/L (SE = 0.0058), respectively, in FT_4_ concentration when analyzed using the natural logarithm scale. In the analysis of the VSR, all air pollutants except for SO_2_ showed a similar pattern. The significant effect of air pollutants on the decrease in FT_4_ concentration was the greatest in the subjects with high VSR [*β* (SE) for each IQR increase = –0.0220 (0.0070) to – 0.0207 (0.0058)] than in the high VAT or high SAT group [*β* (SE) for each IQR increase = – 0.0166 (0.0046) to – 0.0116 (0.0058)]. CO was significantly related to low FT4 concentration in all high adiposity groups, such as high VAT, high SAT, and high VSR groups (all *p* < 0.05).

**Table 4:** Results of stratified analyses by adiposity traits for the association between exposure to air pollution and free thyroxine

Abdominal adiposity	Exposure	FT_4_ (ng/dL)							
		Normal adiposity				High adiposity			
		Crude Model		Adjusted Model^a^		Crude Model		Adjusted Model^a^	
		*β (SE)*	*p*	*β (SE)*	*p*	*β (SE)*	*p*	*β (SE)*	*p*
WC (cm)	Sample *n*	WC < 90 cm (*n* = 1016)				WC ≥ 90 cm (*n* = 1424)			
	PM_10_ (μg/m^3^)	0.0004 (0.0070)	0.9515	− 0.0004 (0.0072)	0.9511	− 0.0113 (0.0059)	0.0530	− 0.0098 (0.0061)	0.1063
	NO_2_ (ppb)	− 0.0097 (0.0062)	0.1168	− 0.0123 (0.0063)	0.0507	− 0.0086 (0.0050)	0.0836	− 0.0085 (0.0051)	0.0919
	SO_2_ (ppb)	− 0.0035 (0.0064)	0.5861	− 0.0036 (0.0064)	0.5713	0.0012 (0.0052)	0.8217	0.0004 (0.0052)	0.9425
	CO (ppm)	− 0.0006 (0.0082)	0.9373	− 0.0024 (0.0083)	0.7757	− 0.0135 (0.0065)	0.0393	− 0.0133 (0.0066)	0.0443
VAT (cm^2^)	Sample *n*	VAT < 100 cm^2^ (*n* = 678)				VAT ≥ 100 cm^2^ (*n* = 1762)			
	PM_10_ (μg/m^3^)	0.0093 (0.0089)	0.2966	0.0107 (0.0092)	0.2471	− 0.0112 (0.0052)	0.0314	− 0.0119 (0.0054)	0.0275
	NO_2_ (ppb)	0.0064 (0.0074)	0.3844	0.0066 (0.0075)	0.3777	− 0.0146 (0.0045)	0.0013	− 0.0166 (0.0046)	0.0004
	SO_2_ (ppb)	0.0093 (0.0079)	0.2390	0.0083 (0.0079)	0.2949	− 0.0044 (0.0047)	0.3558	− 0.0044 (0.0047)	0.3528
	CO (ppm)	0.0029 (0.0101)	0.7728	0.0012 (0.0102)	0.9093	− 0.0119 (0.0060)	0.0456	− 0.0135 (0.0060)	0.0249
SAT (cm^2^)	Sample *n*	SAT < 100 cm^2^ (*n* = 543)				SAT ≥ 100 cm^2^ (*n* = 1897)			
	PM_10_ (μg/m^3^)	− 0.0018 (0.0103)	0.8647	0.0016 (0.0107)	0.8843	− 0.0067 (0.0050)	0.1788	− 0.0071 (0.0052)	0.1719
	NO_2_ (ppb)	− 0.0013 (0.0085)	0.8815	− 0.0012 (0.0087)	0.8909	− 0.0105 (0.0043)	0.0157	− 0.0121 (0.0044)	0.0064
	SO_2_ (ppb)	0.0092 (0.0083)	0.2680	0.0093 (0.0083)	0.2621	− 0.0036 (0.0047)	0.4415	− 0.0041 (0.0047)	0.3790
	CO (ppm)	0.0007 (0.0112)	0.9513	− 0.0000 (0.0112)	0.9966	− 0.0105 (0.0058)	0.0700	− 0.0116 (0.0058)	0.0467
VSR	Sample *n*	VSR < 1.0 (*n* = 1379)				VSR ≥ 1.0 (*n* = 1061)			
	PM_10_ (μg/m^3^)	0.0053 (0.0060)	0.3831	0.0069 (0.0063)	0.2676	− 0.0201 (0.0067)	0.0028	− 0.0220 (0.0070)	0.0017
	NO_2_ (ppb)	0.0000 (0.0053)	0.9960	− 0.0002 (0.0054)	0.9663	− 0.0188 (0.0057)	0.0009	− 0.0207 (0.0058)	0.0004
	SO_2_ (ppb)	0.0055 (0.0057)	0.3315	0.0045 (0.0057)	0.4296	− 0.0066 (0.0058)	0.2612	− 0.0052 (0.0058)	0.3724
	CO (ppm)	0.0012 (0.0070)	0.8689	0.0007 (0.0071)	0.9245	− 0.0190 (0.0076)	0.0123	− 0.0210 (0.0076)	0.0059

FT_4_, free thyroxine; WC, waist circumference; VAT, visceral adipose tissue; SAT, subcutaneous adipose tissue; VSR, visceral-to-subcutaneous fat ratio; PM_10_, particulate matter ≤ 10 μm in diameter; NO_2_, nitrogen dioxide; SO_2_, sulfur dioxide; CO, carbon monoxide; SE, standard error

FT_4_ level was transformed to natural logarithms

The beta coefficient and 95% confidence interval in each air pollutant was scaled to the interquartile range for each pollutant, respectively (9.1 μg/m^3^ for PM_10_, 13.8 ppb for NO_2_, 1.5 ppb for SO_2_, and 0.2 ppm for CO)

^a^The result was adjusted for site of recruitment, age, smoking status (never- or ex- vs. current-smokers), alcohol consumption (never- or ex- vs. current-drinkers), and physical activity (yes vs. no)

## Discussion

We assessed the associations of exposure to air pollution and thyroid hormones, including TSH and FT_4_ concentration, in a Korean population and whether these relations are modified by VAT and SAT areas. We found that persistent exposure to air pollution was significantly related to increased TSH and decreased FT_4_ concentrations. In the stratified analyses according to abdominal adiposity traits, PM_10_, NO_2_, and CO exposure showed a stronger association with TSH and FT_4_ concentrations in the subjects with high adiposity. We identified the significant effects of PM_10_ and CO exposure on thyroid function in all adiposity groups, as measured by WC, VAT and SAT areas, and VSR. Among the abdominal fat-related traits, the impact of air pollution exposure on the thyroid hormone level was the strongest for VSR in the subjects with high adiposity. Our findings suggest that people with high adiposity may be more likely to develop disturbances in thyroid hormones caused by air pollution, especially PM_10_ and CO.

Epidemiological researches have shown associations between exposure to air pollutants and thyroid hormone [5, 6, 8, 9, 26], but most of these studies have shown limited results in pregnant women [5, 6, 9]. A study of children across southern California found that prenatal PM exposure was significantly associated with increased newborn thyroxine concentration [6]. Two epidemiological studies have reported an inverse relationship between PM_2.5_ and NO_2_ and FT_4_ level during pregnancy, although the relationship with TSH level was not significant [5, 9]. A recent cross-sectional, population-based study in China reported that a 10 μg/m^3^ increase in PM_2.5_ exposure decreased 0.12 pmol/L in FT_4_ concentration [26]. Another study reported that exposure to each of the air pollutants including PM_2.5_, PM_10_, NO_2_, and SO_2_ was significantly and linearly associated with the risk of thyroid nodules in Chinese adults [8]. We found that FT_4_ concentration decreased and TSH concentration increased with increasing exposure to air pollutants. Our results were robust even after stratifying according to abdominal adiposity traits. However, these associations require confirmation by prospective cohort studies.

Obesity, especially abdominal adiposity, is closely related to thyroid hormone levels [13]. A recent study of the association between thyroid function and distribution of abdominal adiposity suggests that SAT is independently and positively associated with elevated triiodothyronine (FT_3_) concentration in a China population (male: OR = 0.183, 95% CI = 0.094 to 0.272, and female: OR = 0.089, 95% CI = 0.007–0.171) [22]. Another study also identified the positive association between abdominal subcutaneous fat and TSH or FT_3_ level [27]. In contrast, a cross-sectional study reported that the excessive adipose tissue, particularly VAT, plays the crucial role in thyroid dysfunction [20]. In our study, both VAT and SAT are associated with a decrease of FT_4_ concentration. These different results may be partially explained by several factors, such as study design and population.

With regard to modifying the effect of obese status on the association between exposure to air pollutants and thyroid function, our research team in 2020 provided the first clue that air pollution, including NO_2_ and CO, is strongly linked to thyroid function in people with overweight or obesity [1]. However, it was only a result of showing the modifying effects of overall obesity defined by BMI. To date, to our knowledge, no studies have evaluated the relationship between exposure to ambient air pollution and thyroid functions, considering the abdominal fat distribution in the general population. We found for the first time that the relations between air pollution exposure and thyroid functions were more strongly associated in the subjects with high adiposity, as defined by high VAT, SAT, and VSR areas.

The pathophysiological mechanism linking air pollution, abdominal fat level, and thyroid function is unclear. However, possible hypotheses involving the adipocyte have been proposed. The oxidative stress reaction is the most plausible mechanism. Thyroid hormones regulate the antioxidant system via the promotion of oxidative stress and reactive oxygen species [28, 29]. Both type of hypothyroidism and hyperthyroidism have been shown to be related to oxidative damage to cell structures [28]. The toxic effect of air pollutant exposure also leads to the production of other reactive oxygen species and oxidative injury [30]. Similarly, abdominal fat, particularly the visceral fat accumulation, is responsible for activation of oxidative stress or oxidative injury [18]. In addition, inflammatory response can be a plausible mechanism. Systemic inflammation through cytokine changes induces thyroid dysfunction [28]. Obesity is related to an increased production of cytokines for the pro-inflammatory response (e.g., leptin, TNF-α, and IL-6) and a decreased secretion of adipokines that protect inflammatory response (e.g., adiponectin and IL-10). Such inflammatory cytokines are mainly secreted from visceral adipocytes [18, 19]. Besides, exposure to air pollution is implicated in systemic inflammation [31]. Therefore, our results stratified by VAT or VSR may be interpreted by the synergistic effects of exposure to air pollution and abdominal fat on the oxidative stress or inflammation. However, additional studies are needed to better understand the clear mechanisms.

The strength of our study is the observation, for the first time, that the relationship between air pollution exposure and thyroid hormone is stronger in people with high adiposity, as indicated by WC, VAT and SAT areas, and VSR. Our study has some limitations. First, this study is a cross-sectional type, which cannot be used to identify a causal relation between exposure to air pollutant, abdominal fat distribution, and thyroid hormone. Second, our study did not conduct analysis of possible sex differences in abdominal adiposity distribution because of insufficient data on women. Health outcomes caused by air pollution or obesity may differ between two sexes due to differences in patterns of physical activity, sex hormone levels, occupational features, and the way of life, as well as differences in adiposity distribution. Third, to assess individual air pollution exposure levels, we considered the exposure assessment at the community level using residential postal code districts instead of a more accurate exposure estimate for each participant, because there is not enough relevant data in this population. This method of data collection did not allow us to examine the potential roles of multiple factors, such as occupational characteristics or exposure to air pollution in indoor spaces, residence history, nearness to road traffic conditions, local topography, and change in climate. In this context, each subject's level of exposure to air pollution may have been overestimated or underestimated, which may have contributed to potential misclassification of air pollution exposure. Fourth, it is not possible to determine the temporal consistency between exposure and effects for association analysis. In addition, iodine nutrition, which is known to affect thyroid hormones, could not be included as a confounder due to lack of relevant information. Finally, further research on the short-term effect via lag analysis or association with air pollution in FT_3_ is needed.

## Conclusions

Our data suggest that, in general adult men in Korea, people with high abdominal adiposity may be more vulnerable to increase in TSH and decrease in FT_4_ concentrations when exposed to air pollution. The relationship between air pollution exposure and thyroid hormone seems to differ according to the abdominal adiposity distribution, especially VAT level. Our results provide the first clue that long-term exposure to air pollutants is related to the thyroid hormones in Korean male adults and that the relationship may be modified by abdominal adiposity, especially visceral fat level. Further studies are needed to investigate whether the relationship between air pollution, thyroid hormone, and abdominal fat distribution are causal.

## Data Availability

The data sets generated and analyzed during the current study are not publicly available due to individual privacy, data protection and confidentiality, but are available from the corresponding author on reasonable request.

## Abbreviations

OECD: The Organization for Economic Cooperation and Development
VAT: Visceral adipose tissue
SAT: Subcutaneous adipose tissue
IL-6: Interleukin-6
TNF-α: Tumor necrosis factor-α
TSH: Serum thyrotropin
FT_4_: Free thyroxine
CT: Computed tomography
PM_10_: Particulate matter with an aerodynamic diameter of ≤ 10 μm
NO_2_: Nitrogen dioxide
SO_2_: Sulfur dioxide
CO: Carbon monoxide
WC: Waist circumference
VSR: Visceral-to-subcutaneous fat ratio
IQR: Interquartile range
SEs: Standard errors
